# Effect of High Hydrostatic Pressure Intensity on Structural Modifications in Mealworm (*Tenebrio molitor*) Proteins

**DOI:** 10.3390/foods11070956

**Published:** 2022-03-25

**Authors:** Abir Boukil, Alice Marciniak, Samir Mezdour, Yves Pouliot, Alain Doyen

**Affiliations:** 1 Department of Food Science, Université Laval, Quebec, QC G1V 0A6, Canada; abir.boukil.1@ulaval.ca (A.B.); yves.pouliot@fsaa.ulaval.ca (Y.P.); 2 Institute of Nutrition and Functional Foods (INAF), Université Laval, Quebec, QC G1V 0A6, Canada; 3 Department of Food Science, University of Guelph, Guelph, ON N1G 2W1, Canada; amarcini@uoguelph.ca; 4 AgroParisTech, UMR782 Paris Saclay Food and Bioproduct Engineering, 1 Rue des Olympiades, 91077 Massy, France; samir.mezdour@agroparistech.fr

**Keywords:** high hydrostatic pressure, *Tenebrio molitor*, proteins, structural modification

## Abstract

Processing edible insects into protein extracts may improve consumer acceptability. However, a better understanding of the effects of food processing on the proteins is needed to facilitate their incorporation into food matrices. In this study, soluble proteins from *Tenebrio molitor* (10% *w*/*v*) were pressurized using high hydrostatic pressure (HHP) at 70–600 MPa for 5 min and compared to a non-pressurized control (0.1 MPa). Protein structural modifications were evaluated using turbidity measurement, particle-size distribution, intrinsic fluorescence, surface hydrophobicity, gel electrophoresis coupled with mass spectrometry, and transmission electron microscopy (TEM). The observed decrease in fluorescence intensity, shift in the maximum emission wavelength, and increase in surface hydrophobicity reflected the unfolding of mealworm proteins. The formation of large protein aggregates consisting mainly of hexamerin 2 and ⍺-amylase were confirmed by protein profiles on gel electrophoresis, dynamic light scattering, and TEM analysis. The typical aggregate shape and network observed by TEM after pressurization indicated the potential involvement of myosin and actin in aggregate formation, and these were detected by mass spectrometry. For the first time, the identification of mealworm proteins involved in protein aggregation phenomena under HHP was documented. This work is the first step in understanding the mealworm protein–protein interactions necessary for the development of innovative insect-based ingredients in food formulations.

## 1. Introduction

In order to meet the needs of a consistently growing world population, food production—and more importantly, protein demand—are expected to double by 2050 [1]. As early as 1975, edible insects were suggested as a solution for reducing global food insecurity [2]. Hence, edible insects are gaining interest as a sustainable source of alternative protein [3,4,5]. In addition, from a nutritional point of view, edible insects have a high content (40–75% on dry-matter basis) of good-quality proteins, depending on the insect species [6], and up to 16% of the essential amino acids [7]. Nevertheless, their consumption as whole insects remain limited in Western countries, triggering the emergence of processed insect-protein-based food [8]. The production of insect-protein-based products requires fundamental knowledge of the physicochemical and functional behavior of proteins. Some industrial processes affecting proteins may even improve protein techno-functionalities. Heat treatment, enzymatic hydrolysis, ultrasound, and more recently, high hydrostatic pressure (HHP) have been reported to improve foam capacity, solubility, water-holding capacity, and other desirable qualities [9,10,11]. More specifically, HHP is a nonthermal process that applies isostatic pressure (up to 1000 MPa), inducing the destabilization of noncovalent bonds (hydrophobic, hydrogen, and ionic bonds) within the protein structure. High hydrostatic pressure generally has no impact on the covalent bonds of proteins, but induces disulfide bond exchange, especially when proteins with free thiols are pressurized [12,13]. Thus, HHP could induce the formation of new inter- or intramolecular disulfide bonds (formed between cysteine residues), followed by protein aggregation [14]. Consequently, the secondary, tertiary, and quaternary structures of pressurized proteins undergo different conformational modifications, leading to new protein–protein interactions, depending on the pressure level [15].

Among edible insects, mealworm (*Tenebrio molitor*) is one of the most popular species as a food resource. Mealworm typically contains 53% protein (dry basis), consisting of a very large diversity of proteins, a detailed profile of which was recently determined by using shotgun proteomics approaches [16]. However, its major proteins consist of fibrous proteins [17] (tropomyosin, myosin, twitchin, actin), but also hemolymph proteins (hexamerin 1 and 2) and enzymes (α-amylase, arginine kinase, prophenoxidase) [18,19]. More specifically, hexamerin 2 is composed of two free thiols (UniProtKB-Q95PI7), whereas ⍺-amylase is stabilized by the presence of four disulfide bonds (UniProtKB-P56634). Although recent reviews have been published that discussed the impact of conventional and emerging food processes on edible insect protein extraction, purification, and techno-functionalities [20,21], the impact of HHP on the structure of edible insect proteins remains poorly documented. To the best of our knowledge, only Kim et al. demonstrated that HHP improved the techno-functional properties of proteins extracted from *Protaetia brevitarsis seulensis* [22], whereas Ugur et al. (2020) and Bolat et al. (2021) evaluated the impact of HHP-assisted extraction on the physicochemical properties of oils extracted and edible insect powder generated from *Acheta domesticus* and *T. molitor* [23,24]. Consequently, the aim of this work was to investigate the effect of HHP treatment intensity on the conformational changes and aggregation behavior of mealworm protein extract. More specifically, the objectives were: (1) to determine the impact of HHP on protein structural changes by evaluating the modification in turbidity of mealworm solutions under pressure treatments, as well as changes in particle size, intrinsic fluorescence, and surface hydrophobicity; and (2) to compare protein profiles of control and pressure-treated mealworm protein extracts and to determine the mealworm proteins involved in aggregate formation.

## 2. Materials and Methods

### 2.1. Raw Material

Three different batches of living mealworm larvae were kindly provided by Groupe Neoxis (Saint-Flavie, Québec, Canada). First, larvae were separated from the feed substrate and frass residues by passing them through an 800 μm sieve. Next, living mealworm larvae were killed by freezing at −20 °C overnight and then freeze-dried. Finally, the freeze-dried larvae were ground into a powder (Thermomix^®^, Vorwerk, Wuppertal, Germany).

### 2.2. Methods

#### 2.2.1. Soluble Protein Recovery

**Figure 1:**
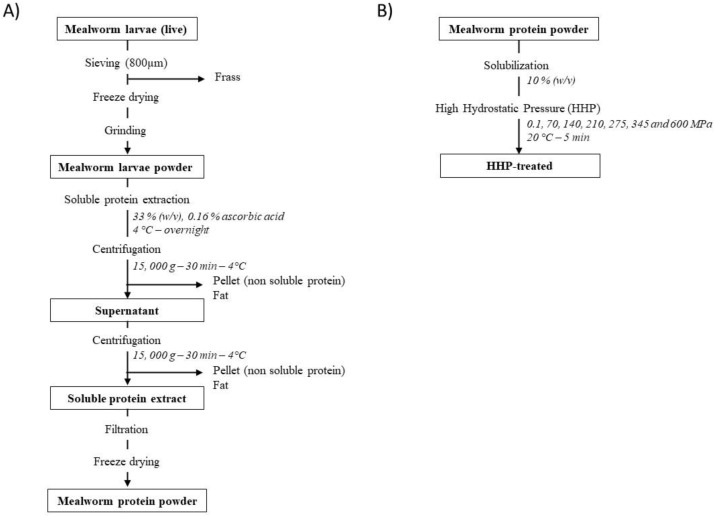
Experimental design of the production of mealworm protein extract (**A**) and its treatment by high hydrostatic pressure (**B**).

Soluble proteins of mealworm larvae were extracted and recovered from each batch as described by Yi et al. (2013) with the following modifications [25]. The mealworm powder (400 g) obtained after freeze-drying and grinding was suspended in deionized water (1200 mL), and mixed with ascorbic acid (2 g) to prevent oxidation and enzymatic browning. The suspension was stirred overnight at 5 °C and centrifuged at 15,000× *g* for 30 min at 4 °C. The supernatant containing the soluble insect components was recovered and centrifuged again at 15,000× *g* for 30 min at 4 °C for optimal separation of fat and soluble and insoluble insect compounds from the proteins. The supernatant was collected and vacuum-filtered through Whatman No. 1 filter paper to retain any residual insoluble particles. Finally, the filtrate was collected, freeze-dried, and stored at −20 °C until further use. The detailed approach used to generate the mealworm protein powder is illustrated in Figure 1A.

#### 2.2.2. Proximate Composition of Mealworm Protein Extracts

Total crude protein content of the freeze-dried powder was determined using the Kjeldahl method according to AOAC method 928.08 (AOAC International, 2012). The nitrogen conversion factor was 5.60 for the larvae protein extract, as determined by Janssen et al. (2017) [6]. Moisture and ash contents were determined by AOAC 925.09 and 923.03 methods, respectively. The crude fat content was obtained after hexane extraction based on a Soxhlet method (AOAC 960.39). The proximate composition of the mealworm protein powder, determined on a dry basis, was 56.45 ± 0.02% protein, 0.51 ± 0.00% fat, and 7.33 ± 0.57 ash. The moisture content was 10.33 ± 0.57%.

#### 2.2.3. High Hydrostatic Pressure Treatments of Mealworm Proteins

Mealworm protein powder was suspended in deionized water at a protein content of 10% (*w*/*v*) and stirred overnight at 4 °C prior to HHP treatments (Figure 1B). Then, 50 mL of each sample was transferred into flexible plastic bags to be pressure-treated at 70, 140, 210, 275, 345, and 600 MPa for 5 min at 20 °C in a discontinuous hydrostatic pressurization unit (Hiperbaric 135, Hiperbaric, Burgos, Spain), using water as a pressure-transmission medium. The pressurization rate was 27.5 s for 100 MPa and the decompression was instantaneous. Pressurization values were previously determined (data not shown) using a high-pressure cell directly connected to a photon-counting spectrofluorometer (ISS Inc., Champaign, IL, USA). Non-pressurized protein extract suspensions (0.1 MPa) were used as controls.

### 2.3. Analysis

#### 2.3.1. Turbidity Measurement

The turbidity of control (0.1 MPa) and pressure-treated mealworm protein solutions (10% *w*/*v*) was measured by spectrophotometry at 595 nm (Thermo Labsystems Multiskan Spectrum Microplate UV–vis reader, Thermo Fisher Scientific, Waltham, MA, USA). First, protein solutions were diluted 1:16 with deionized water. A volume of 200 µL of each protein solution diluted 1:16 was loaded into transparent 96-well microplates (Greiner Bio-One, Kremsmuünster, Austria). The turbidity measurements were reported as optical density at 595 nm (OD_595_), and deionized water was used as a blank sample. Turbidity measurements of all samples and blanks were performed in triplicate, and one measurement was taken per replicate.

#### 2.3.2. Particle-Size Measurement

Particle-size distribution in the control and pressure-treated mealworm protein solution (10% *w*/*v*) was measured by laser light scattering using a Mastersizer 3000 analyzer (Malvern Mastersizer 3000, Malvern Instruments, Malvern, UK). The results were analyzed with Mastersizer 3000 software. Particle and dispersant (i.e., water) refractive indexes were set at 1.48 and 1.33, respectively. Measurements were taken with two laser sources at 632.8 and 470 nm. Samples were directly diluted in the measurement cell of the instrument (Mastersizer 3000) to reach 5% obscuration [26].

#### 2.3.3. Intrinsic Fluorescence Spectroscopy Measurements

Tryptophan is known to emit intrinsic fluorescence that is measurable by fluorescence spectroscopy. Changes in emission spectra from tryptophan can be seen in response to protein conformational change or denaturation [27]. A photon-counting spectrofluorometer (ISS Inc., Champaign, IL, USA) was used to evaluate the state of denaturation of the mealworm protein solutions (10% *w*/*v*) after pressure treatments. The control and the pressure-treated mealworm protein solutions (10% *w*/*v*) were diluted 1:50 (0.2% protein) and loaded in a quartz cuvette at room temperature. The intrinsic fluorescence of each mealworm protein extract suspension was measured using an excitation wavelength of 280 nm, and fluorescence emission spectra were recorded between 300 and 500 nm [28]. All fluorescence measurements were done in triplicate.

#### 2.3.4. Surface Hydrophobicity

The surface hydrophobicity of the control (0.1 MPa) and pressure-treated mealworm proteins was determined using 1-anilino-8-naphtalenesulfonate (ANS) and measured according to Nakai (2003) [29] with slight modifications. The mealworm protein solutions (10% *w*/*v*) were diluted with 2 mM phosphate buffer at pH 7 to reach 0.1% protein (*w*/*v*), stirred for 60 min at room temperature, and then kept at 5 °C overnight. These solutions were centrifuged (10,000× *g*, 20 min, 18 °C), and protein content in the supernatant (soluble protein) was determined using the Dumas method (Elementar Rapid Micro N Cube, Langenselbold, Germany), with a nitrogen-to-protein conversion factor of 5.60, as proposed by Janssen et al., 2017 [6]. Supernatants were diluted with 2 mM phosphate buffer (pH 7) to a concentration of 0.01 to 0.05% (*w*/*v*) and stirred at room temperature for 10 min. Aliquots of the ANS solution were prepared (8 mM in 0.1 M phosphate buffer, pH 7), and 15 μL was added to 3 mL of each of the diluted mealworm protein solutions (0.01 to 0.05% (*w*/*v*)). The different solutions were then vortexed and equilibrated in the dark for 5 min. Fluorescence intensity was measured using a quartz cell (1 cm path length) in a spectrofluorometer (ISS Inc., Champaign, IL, USA) at excitation and emission wavelengths of 380 and 480 nm, respectively [30]. Surface hydrophobicity was calculated by linear regression, and was determined to be the initial slope for fluorescence intensity versus protein concentration. Each spectrum was blank-corrected, and experiments were performed in triplicate for each sample.

#### 2.3.5. Transmission Electron Microscopy

Transmission electron microscopy (TEM) was performed on pressure-treated and control protein solutions (10% *w*/*v*) prepared according to the protocol described by Marciniak et al. (2018) [31]. A droplet of pressure-treated or control sample was added to 3% uranyl acetate on Formvar-film-coated 200 mesh nickel grids and dried in air. A JEOL JEM-1230 TEM (Tokyo, Japan) operating at 80 kV was used for imaging. A Gatan US1000SP1 ultrascan camera (Gatan, Inc., Pleasanton, CA, USA) was used for image capture, and the images were analyzed using Gatan DigitalMicrograph 2.11 software.

#### 2.3.6. Determination of Protein Profiles of Control and Pressure-Treated Mealworm Protein Extracts

Protein profiles of the control and pressure-treated mealworm protein solutions (10% *w*/*v*) were obtained by native and sodium dodecyl sulphate–polyacrylamide gel electrophoresis (SDS-PAGE) under reducing conditions. First, the protein-aggregation states of both the control and pressure-treated samples were determined using PAGE under native conditions with a 4–20% TGX Stain-Free polyacrylamide gel (Bio-Rad, Mississauga, ON, Canada), as described by Boukil et al. (2018) with a slight modification [15]. Twenty-five microliters of pressure-treated protein solutions were diluted 1:8 with distilled water. Twenty-five microliters of each diluted protein solution was mixed with the same volume of native sample buffer. Then, 10 µL of each sample was loaded into the gel wells in triplicate. For SDS-PAGE, the samples were diluted as described above, using a reducing sample buffer (5% 2-mercaptoethanol, 95% Laemmli buffer (Bio-Rad, Mississauga, ON, Canada)). Then, the diluted protein samples were immersed in a boiling water bath for 10 min and cooled before loading 10 µL of each sample in the gel wells. The running buffer for native electrophoresis was composed of 20% methanol, 10% Tris-glycine buffer, and 70% deionized water, while the running buffer for the reducing electrophoresis was composed of 10% Tris-glycine SDS buffer and 90% deionized water. Electrophoresis was performed using 4–20% TGX Stain-Free polyacrylamide gel (Bio-Rad, Mississauga, ON, Canada) at 15 mA for 1 h at room temperature. Proteins were stained with Coomassie blue (1 g/L of Coomassie Brilliant Blue R-250, 10% acetic acid, and 40% ethanol) and destained with a solution of 10% (*v*/*v*) methanol and 10% (*v*/*v*) acetic acid. The molecular weights of the mealworm proteins were estimated using a molecular weight standard (Precision Plus Protein™ 161-0373 All Blue Prestained Protein Standards, Bio-Rad, Mississauga, ON, Canada). Images of the gels were captured using a ChemiDoc™ MP Imaging System (ChemiDoc MP, Bio-Rad, Mississauga, ON, Canada).

#### 2.3.7. Protein Identification by Mass Spectrometry

Mass spectrometry (MS) analysis was performed by the Proteomics Platform of the Research Center of the Centre Hospitalier Universitaire (CHU) of Quebec City (QC, Canada). Relevant bands observed in PAGE were excised and washed with Milli-Q water. The MS analysis was performed as described by Boukil et al., 2020 [10]. Contaminants were detected and protein sequences were aligned using Uniprot databases and the Tenebrionidae family (24,496 entries) database.

### 2.4. Statistical Analysis

Proximate composition and analysis were performed in triplicate for each sample. All the data were analyzed as a randomized complete block design using the GLM procedure of SAS (University Edition, SAS^®^ 3.8 software, Cary, NC, USA). The results were expressed as mean ± standard deviation (SD). Turbidity, particle-size distribution, intrinsic fluorescence, and surface hydrophobicity data were subjected to a one-way ANOVA, multiple comparison statistical analysis using the Statistical Analysis System (SAS) University Edition, SAS^®^ 3.8 software. Tukey tests (α = 0.05) were used as a multiple-comparison test.

## 3. Results

### 3.1. Change in Turbidity of Mealworm Proteins under HHP

**Figure 2:**
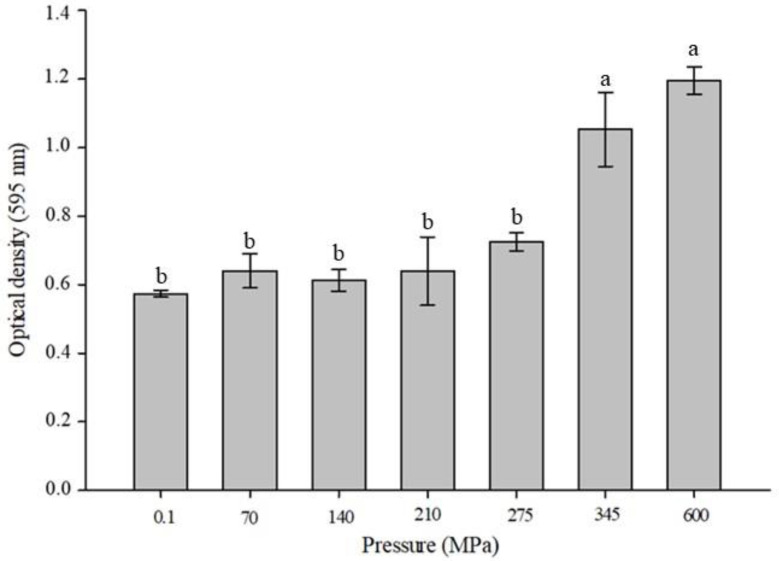
Optical density (OD_595_ nm) of control (0.1 MPa) and pressure-treated (70–600 MPa for 5 min) mealworm protein samples at 10% (*w*/*v*). The data represent mean values of triplicates ± standard deviation. Multiple means comparisons were performed between pressure treatments (one-way ANOVA, multiple comparison, Tukey test, ⍺ = 0.05). Different lowercase letters (a and b) indicate mean values with significant differences.

The turbidity values for mealworm protein samples (10% (*w*/*v*)) were compared before (control) and after pressurization from 70 to 600 MPa (Figure 2). The turbidity measurements highlighted some interesting differences, since the OD_595_ increased from 0.573 (±0.011–0.1 MPa) to 1.196 (±0.040–600 MPa). Statistical analysis revealed similar OD_595_ values at 345 and 600 MPa (*p* > 0.05) that were significantly higher than those obtained for the other pressurization conditions (*p* < 0.0001).

### 3.2. Particle-Size Distribution of Mealworm Proteins under HHP

**Figure 3:**
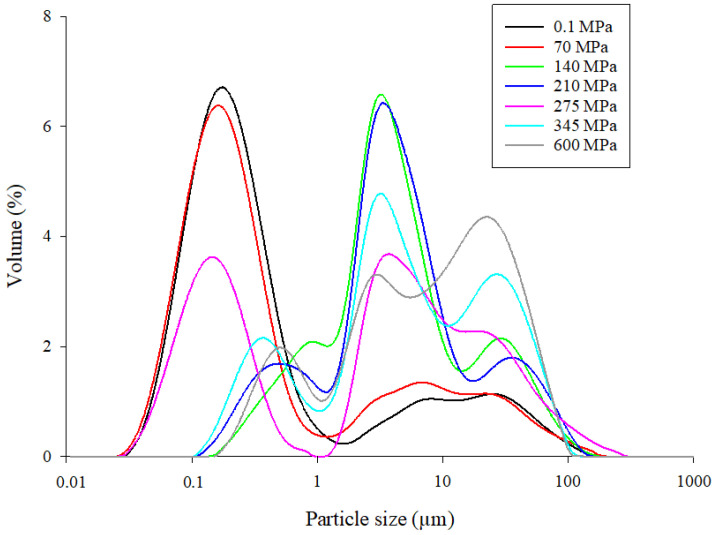
Particle-size distribution of control (0.1 MPa) and pressure-treated (70–600 MPa for 5 min) mealworm protein solutions.

Figure 3 shows the particle-size distribution of the control (0.1 MPa) and pressure-treated (70–600 MPa for 5 min) mealworm protein samples. Two different particle-size populations were obtained for the control (0.1 MPa) and pressure-treated mealworm proteins at 70 MPa, with the first population consisting of particle sizes ranging from 0.03 to 1.5 µm, and the second consisting of particle sizes from 1.0 to 200.0 μm. Three different particle-size populations were observed from 140 to 600 MPa. At 275 MPa, the first particle-size population (0.1 to 1.5 µm) was quite similar to the one obtained at 0.1 and 70 MPa, except that a lower percent volume was obtained. For the other pressurization conditions (140, 210, 345, and 600 MPa), the particle sizes of the first population (0.1 µm to 1.5 µm) increased compared to 0.1, 70, and 275 MPa, whereas the percent volumes decreased. The large second particle-size population observed at 0.1 and 70 MPa (1.0 to 200.0 μm) was divided into two distinct populations with similar particle sizes, ranging from 1.0 to 20 µm and 10 to 300 µm for mealworm samples treated at pressures of 140 to 600 MPa.

### 3.3. Modification of Mealworm Protein Intrinsic Fluorescence after HHP Treatment

**Figure 4:**
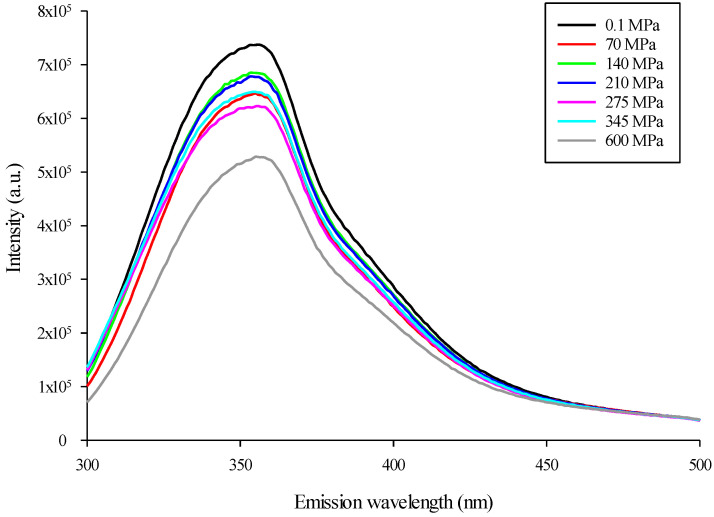
Intrinsic fluorescence spectra of control (0.1 MPa) and pressure-treated (70 to 600 MPa) mealworm protein solutions.

The conformational modifications of the control and pressure-treated mealworm proteins were assessed by monitoring changes in the intrinsic fluorescence emission of the tryptophan indole group between 300 and 500 nm [32]. As observed in Figure 4, and for all pressures applied, the maximum intensity of the fluorescence emission of pressure-treated mealworm proteins was lower than the control (0.1 MPa). More specifically, pressurization of mealworm proteins at 600 MPa induced the largest decrease in fluorescence intensity (~30%), whereas similar decreases were obtained from 70 to 345 MPa at an average of 15%, compared to the control. Moreover, compared to the control (0.1 MPa-353 nm), the maximum emission wavelength at 600 MPa was redshifted to a slightly higher value (355 nm).

### 3.4. Surface Hydrophobicity of HHP-Treated Mealworm Proteins

Surface hydrophobicity (H_0_) is the measure used to evaluate modification of protein conformation. ANS binds to the hydrophobic sites of proteins, reflecting their capacity to undergo structural modification [33,34]. The changes in surface hydrophobicity of pressure-treated mealworm protein solutions are shown in Table 1. The surface hydrophobicities of the control (0.1 MPa) and pressure-treated mealworm proteins at 70, 140, and 210 MPa were similar (*p* > 0.05), with values ranging from 1.50 to 1.80 × 10^6^. However, the surface hydrophobicities of mealworm protein solutions receiving HHP treatments from 275 to 600 MPa (2.41 to 2.33 × 10^6^) were significantly greater (*p* < 0.05) than those of the control (1.50 × 10^6^). In addition, similar surface hydrophobicity values were obtained for HHP treatments from 275 to 600 MPa (*p* > 0.05).

### 3.5. Protein Profile and Identification of Pressure-Induced Protein Aggregates

**Figure 5:**
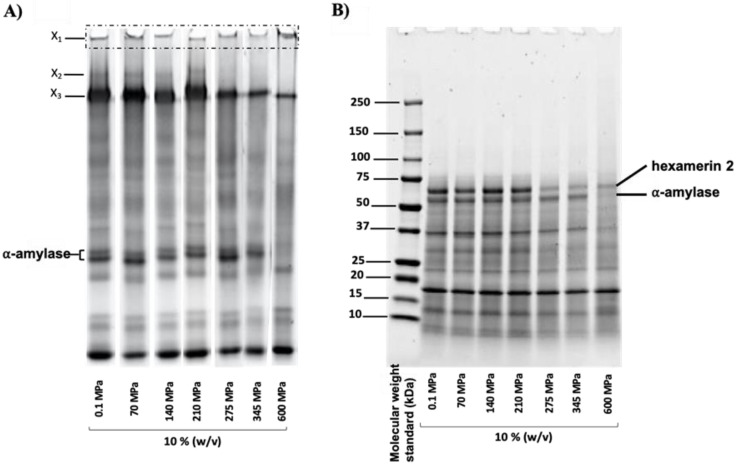
Native (**A**) and denatured/reduced (**B**) PAGE of control (0.1 MPa) and pressure-treated mealworm proteins. The proteins composing bands X_1_ and X_2_, as well as ⍺-amylase and hexamerin 2, were identified by proteomic analysis.

Mealworm protein profiles before (control or 0.1 MPa) and after pressurization were also analyzed using PAGE under native conditions (Figure 5A), and under denaturing and reducing conditions (SDS-PAGE—Figure 5B). Complementarily, and considering the more drastic impact of HHP at 600 MPa, Table 2 presents the composition of excised bands X_1_ obtained from the native polyacrylamide gel for samples at 0.1 MPa (control) and 600 MPa only.

The native PAGE of the pressurized mealworm proteins (Figure 5A) showed the presence of proteins (X_1_) trapped in the loading wells for all pressurization conditions, but with the greatest intensity at 600 MPa. According to the mass spectrometry analysis (Table 2), this population (X_1_) was mainly composed of twitchin at 0.1 MPa (control); whereas at 600 MPa, it was composed of ⍺-amylase and hexamerin 2. In addition, the total spectrum counts of twitchin decreased from 230 to 79 after pressure treatment, whereas those of ⍺-amylase and hexamerin 2 increased from 28 to 252 for hexamerin 2 and from 4 to 122 for ⍺-amylase. Muscle proteins (actin and myosin) were also detected in the wells of native gels in the control and 600 MPa pressure-treated mealworm protein samples. Total spectrum counts of actin increased from 18 to 43 after pressure treatment, whereas those of myosin decreased from 17 to 5.

In parallel, bands corresponding to ⍺-amylase disappeared from the gel after treatment at 600 MPa, whereas above 275 MPa, the intensities of X_3_ bands decreased and X_2_ bands (detected as hexamerin 2 by proteomic analysis) disappeared. Under reducing conditions (Figure 5B), protein profiles were similar for the control and pressurized samples, with the exception of the hexamerin 2 and ⍺-amylase proteins (identified by mass spectrometry). In fact, similar to observations in the native PAGE for X_2_ (Figure 5A), the intensity of the band corresponding to hexamerin 2 decreased drastically for pressures above 275 MPa. In addition, the band corresponding to ⍺-amylase disappeared at 600 MPa, as observed in the native PAGE (Figure 5A).

### 3.6. Microstructure of Pressure-Treated Mealworm Proteins

**Figure 6:**
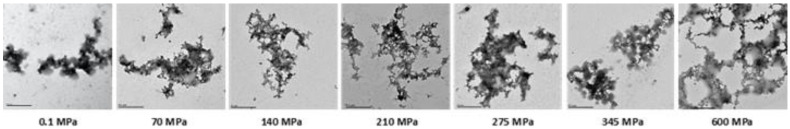
TEM images of control (0.1 MPa) and pressure-treated mealworm protein samples. Magnification factor: 10 K; observation scale: 0.5 µm.

To further investigate the impact of HHP on the structure and aggregation state of mealworm proteins, the control and pressurized samples were analyzed by negative-stained TEM, as presented in Figure 6.

The TEM images revealed differences in the shapes of particles between the control and pressurized samples, especially at 600 MPa. At 0.1 MPa, proteins were packed into dense and globular aggregates. However, as the pressure level increased, irregular shaped, porous particles were formed (70 to 345 MPa) with a large, thin and amorphous network specifically visible under severe pressure (600 MPa).

## 4. Discussion

The aim of this work was to investigate the impact of HHP on the modification of protein structure and profile. Overall, HHP induced an increase in the turbidity and particle-size distribution of the mealworm protein solutions. Moreover, the decrease and shift in the fluorescence intensity observed for pressure-treated mealworm proteins could be attributed to protein unfolding, mainly ⍺-amylase and hexamerin 2, which correlated with the increase in surface hydrophobicity of proteins treated at high pressure levels. Finally, the microstructure analysis showed the formation of a wide and porous protein network, especially at 600 MPa.

### 4.1. Impact of High Hydrostatic Pressure on Mealworm Protein Structural Changes

Protein denaturation, and to a larger extent, protein structural change, is usually assessed by measuring the turbidity or optical density (OD) at 595 nm [36]. More specifically, an increase in OD generally correlated to an increase in the formation of protein aggregates [37], as previously demonstrated for pressure-treated marine [38], milk [39], egg [40], and soy [41] proteins, as well as mixed protein systems [42]. Consequently, the increase in turbidity observed in pressure-treated mealworm protein solutions at 345 MPa could indicate the formation of protein aggregates (Figure 2). An increase in turbidity of a protein solution is generally correlated with an increase in particle-size distribution [43]. Thus, to obtain further evidence of structural modifications in mealworm protein solutions, the change in particle-size distribution was evaluated between the control (0.1 MPa) and pressure-treated mealworm protein solutions. High hydrostatic pressure had an obvious influence on the particle-size distribution, since the average particle size of the mealworm protein solutions treated at 140 to 600 MPa consistently increased compared to the control and the lower pressure level of 70 MPa (Figure 3). This kind of increase in particle-size distribution after HHP treatment was observed previously for different proteins [44,45], and was mainly explained by protein–protein interactions, particularly intermolecular disulfide bond formation and protein aggregate generation [46,47,48]. Consequently, the shift in particle-size distribution toward large particle sizes after pressure treatment indicated the formation of mealworm protein aggregates, confirming the results of the turbidity analysis. Additionally, conformational changes accompanying HHP treatment of mealworm protein solutions were monitored by measuring the fluorescence intensity of tryptophan. The change in the intensity of fluorescence emission and concomitant shift in the maximum emission wavelength correlated with the exposure of the tryptophan residues to an aqueous environment, and was used to monitor unfolding/refolding of proteins [49]. After pressurization and compared to the control, the intensities of the mealworm proteins’ fluorescence emissions decreased, and were highest at 600 MPa. However, and surprisingly, no tendencies were observed between pressurization level and emission spectra intensities except at 600 MPa, which could suggest some resistance of the proteins to HHP. More specifically, compared to the control (0.1 MPa), the maximum emission wavelength (µmax) at 600 MPa was redshifted to a slightly higher value (Figure 5). A decrease and redshift in fluorescence intensity indicated that the tryptophan residues of the mealworm proteins were exposed to a polar microenvironment [50]. Therefore, these results suggested that HHP resulted in protein unfolding, exposing tryptophan residues to a more polar environment than in the native mealworm proteins [51,52]. In addition to intrinsic fluorescence intensity, surface hydrophobicity of proteins was another structural characteristic used to evaluate changes in protein conformation [52]. An increase in surface hydrophobicity was related to the exposure of the side chain of an aromatic amino acid. Consequently, the higher the surface hydrophobicity, the greater the number of hydrophobic groups exposed to the outside of the proteins [53]. The surface hydrophobicities of the mealworm proteins following HHP treatments from 275 to 600 MPa were higher than that of the control (0.1 MPa), as well as the pressure-treated solutions at 70, 140, and 210 MPa (Table 1). Increases in surface hydrophobicity of different proteins treated with HHP was also observed by several authors, indicating the proteins’ partial denaturation and aggregation [53,54,55]. Although no literature was available regarding the impact of HHP on the surface hydrophobicity of mealworm proteins, it was demonstrated that heating [56] and ultrasound [57] treatments increased the surface hydrophobicities of mealworm protein extract and meal, respectively. The findings regarding mealworm protein structural modifications should be further studied to characterize the proteins involved in the aggregation phenomenon.

### 4.2. Effect of Pressurization on Protein Profiles and Determination of Proteins Involved in Aggregate Formation

Native PAGE (Figure 5A) showed the presence of proteins trapped in the loading wells, especially at 600 MPa, that were identified as twitchin, hexamerin, and ⍺-amylase. The detection of twitchin in gel electrophoresis wells was probably due to its high molecular weight (995 kDa). Under reducing conditions (Figure 5B), ⍺-amylase was the most affected by pressurization above 275 MPa, followed by hexamerin 2. Consequently, it was possible that these two main proteins were involved in the formation of protein aggregates under HHP. Indeed, structurally, hexamerin 2, which is the non-copper-and-oxygen binding form of hemocyanin, is composed of two free thiols (Cys289 and Cys13), while ⍺-amylase is stabilized by the presence of four disulfide bonds (Cys28-Cys84, Cys134-Cys148, Cys354-Cys360, and Cys427-Cys437). It has already been shown that the presence of a free thiol group in a protein solution was sufficient to enhance the denaturation of proteins with higher stability (through the presence of disulfide bonds), as has been observed in other matrices such as in milk and whey for the interaction of β-lactoglobulin (which contains a free reactive thiol) and α-lactalbumin (no free thiol) under HHP [58]. In fact, the intake of energy through HHP treatment exposed buried thiol groups, and thus triggered potential inter- and intramolecular protein–protein interactions [59]. Our results agreed with the literature on the impact of HHP on similar proteins from various sources. Hexamerin 2, described as a storage protein in insects, is composed of the three hemocyanin (N, M, and C) domains. Until now, the literature has focused on the effect of pressure on hemocyanin (the oxygen-binding form mainly found in mollusks) [60,61], and has demonstrated structural modification and protein denaturation with increasing pressure, especially above 400 MPa. As a result, the authors were able to demonstrate a decrease in the allergenicity of hemocyanin from squid under HHP [62]. In addition, Reinhart et al. (1993) showed that an HHP treatment of 200 MPa was sufficient to dissociate the hexameric form of the hemocyanin [63].

Alpha-amylase is found in various sources, from insects to microorganisms and plants. While no literature on the effect of HHP on ⍺-amylase from insects was available, a few studies have focused on other sources, and their results supported our observations. For instance, Grauwet et al., 2009 demonstrated that a 600 MPa, 10 min HHP treatment decreased the activity of the ⍺-amylase from *Bacillus subtilis* down to 35–0% (10–50 °C) [64]. Similarly, ⍺-amylase from malt barley was drastically impacted by HHP treatment at 600 MPa for 10 min, with total loss of activity at 800 MPa [65].

Actin and myosin were also detected in native gel wells after pressurization at 600 MPa (Table 2) but in a lower amounts than twitchin, hexamerin, and α-amylase. As reported in the literature, mealworm proteins consist of fibrous proteins (muscle proteins) [17], hemolymph proteins (hexamerin 1 and 2), and enzymes (α-amylase, arginine kinase, prophenoxidase) [18,19]. Fibrous proteins are known to possess a highly ordered structure stabilized by many hydrogen bonds, with very little tertiary and quaternary structure [66]. In contrast to globular proteins, fibrous proteins are less impacted by HHP, principally due to their very compact structure and the high number of hydrogen bonds [67,68], which explains their low detection in native gel wells. However, the presence of actin and myosin may have promoted the formation of aggregates. While no literature was available regarding the impact of HHP on mealworm proteins’ conformational structures, it was recently demonstrated that aggregation of mealworm myosin heavy chains, probably caused by the formation of intermolecular S–S bonds, was induced by frozen storage of lesser mealworm larvae [25]. Some authors also showed that myosin from tilapia was affected by HHP at 200 MPa, with the formation of myosin aggregates [69,70]. Finally, Hsu et al. (2007) showed that tilapia actomyosin aggregates were formed at pressures above 200 MPa due to disulfide bonds [71]. The involvement of muscle proteins in the formation of aggregates was also supported by microstructure results. Indeed, visual inspection of the micrographs showed a more open structure with some level of protein aggregation for pressure-treated samples when compared with the denser structure of the control (0.1 MPa) mealworm protein samples. Similar microstructures in terms of aggregate shapes and porous networks generated were published by Hsu et al. (2007) after HHP treatments of tilapia actomyosin at pressures up to 250 MPa [71]. According to these authors, at pressures up to 250 MPa, actomyosin filaments were shortened by pressure treatment, probably due to the dissociation of myosin subunits and depolymerization of actin. The evolution of the microstructure observed until 600 MPa could be a result of a similar mechanism on muscular mealworm proteins, thus confirming the detection of actin and myosin by MS analysis.

## 5. Conclusions

The results generated in this work confirmed that mealworm protein structures were modified after HHP treatments, mainly at 600 MPa, with the formation of high-molecular-weight protein aggregates. These aggregates were shown to be specifically composed of twitchin, ⍺-amylase, and hexamerin 2. Actin and myosin were also involved in the formation of aggregates, which was confirmed by the specific protein network shape observed by microscopy. Further investigations are necessary to determine the interactions involved in the formation of aggregates, and to characterize the impact of HHP on techno-functional properties of mealworm protein extract for possible uses as innovative ingredient in food formulations.

## Figures and Tables

**Table 1:** Surface hydrophobicity (H_0_) of control (0.1 MPa) and pressure-treated (70–600 MPa for 5 min) mealworm protein solutions.

Pressure Level (MPa)	H_0_ (Slope *×* 10^6^) *
0.1	1.50 ± 0.03 ^b^
70	1.84 ± 0.14 ^b^
140	1.58 ± 0.20 ^b^
210	1.80 ± 0.11 ^b^
275	2.41 ± 0.01 ^a^
345	2.33 ± 0.02 ^a^
600	2.33 ± 0.02 ^a^

* Results are given as the mean ± standard deviation. Multiple means comparisons were performed between pressure treatments (Tukey test, ⍺ = 0.05). Different letters (a and b) indicate mean values with significant differences.

**Table 2:** Proteomic analysis of native PAGE wells (X_1_) of control (0.1 MPa) and HHP-treated (600 MPa) mealworm protein solutions.

Identified Proteins	UniProt ID	MW (kDa)	Total Spectrum Count (TSC) ^1^	
			0.1 MPa	600 MPa
**Twitchin** OS = *Asbolus verrucosus* OX = 1,661,398 GN = BDFB_000398 PE = 4 SV = 1	A0A482W446_9CUCU	995	230	79
**Hexamerin 2** OS = *Tenebrio molitor* OX = 7067 PE = 2 SV = 1	Q95PI7_TENMO	85	28	252
**Alpha-amylase** OS = *Tenebrio molitor* OX = 7067 PE = 1 SV = 1	AMY_TENMO	51	4	122
**Myosin heavy chain**, muscle isoform X13 OS = *Asbolus verrucosus* OX = 1,661,398 GN = BDFB_000378 PE = 3 SV = 1	A0A482VBZ5_9CUCU	256	17	5
**Actin-87E-like Protein** OS = *Tribolium castaneum* OX = 7070 GN = TcasGA2_TC003326 PE = 3 SV = 1	D6WF19_TRICA	42	18	43

^1^ TSC, defined as the total number of spectra identified for a protein, is a semiquantitative measure of the abundance of a given protein [10,35].

## Data Availability

Data are contained within the article.

## References

[1] Van Huis A., Van Itterbeeck J., Klunder H., Mertens E., Halloran A., Muir G., Vantomme P. (2013). Edible Insects: Future Prospects for Food and Feed Security.

[2] Meyerrochow V.B. (1975). Can Insects Help to Ease Problem of World Food Shortage. Search.

[3] Hall F.G., Jones O.G., O’Haire M.E., Liceaga A.M. (2017). Functional Properties of Tropical Banded Cricket (*Gryllodes sigillatus*) Protein Hydrolysates. Food Chem..

[4] Hall F., Johnson P.E., Liceaga A. (2018). Effect of Enzymatic Hydrolysis on Bioactive Properties and Allergenicity of Cricket (*Gryllodes Sigillatus*) Protein. Food Chem..

[5] Purschke B., Brüggen H., Scheibelberger R., Jäger H. (2018). Effect of Pre-Treatment and Drying Method on Physico-Chemical Properties and Dry Fractionation Behaviour of Mealworm Larvae (*Tenebrio molitor* L.). Eur. Food Res. Technol..

[6] Janssen R.H., Vincken J.-P., van den Broek L.A., Fogliano V., Lakemond C.M. (2017). Nitrogen-to-Protein Conversion Factors for Three Edible Insects: *Tenebrio Molitor*, Alphitobius Diaperinus, and Hermetia Illucens. J. Agric. Food Chem..

[7] Wu R.A., Ding Q., Yin L., Chi X., Sun N., He R., Luo L., Ma H., Li Z. (2020). Comparison of the Nutritional Value of Mysore Thorn Borer (*Anoplophora chinensis*) and Mealworm Larva (*Tenebrio molitor*): Amino Acid, Fatty Acid, and Element Profiles. Food Chem..

[8] Tan H.S.G., Fischer A.R., Tinchan P., Stieger M., Steenbekkers L.P.A., van Trijp H.C. (2015). Insects as Food: Exploring Cultural Exposure and Individual Experience as Determinants of Acceptance. Food Qual. Prefer..

[9] Stone A.K., Tanaka T., Nickerson M.T. (2019). Protein Quality and Physicochemical Properties of Commercial Cricket and Mealworm Powders. J. Food Sci. Technol..

[10] Boukil A., Perreault V., Chamberland J., Mezdour S., Pouliot Y., Doyen A. (2020). High Hydrostatic Pressure-Assisted Enzymatic Hydrolysis Affect Mealworm Allergenic Proteins. Molecules.

[11] Dion-Poulin A., Laroche M., Doyen A., Turgeon S.L. (2020). Functionality of Cricket and Mealworm Hydrolysates Generated after Pretreatment of Meals with High Hydrostatic Pressures. Molecules.

[12] Mozhaev V.V., Heremans K., Frank J., Masson P., Balny C. (1994). Exploiting the Effects of High Hydrostatic Pressure in Biotechnological Applications. Trends Biotechnol..

[13] Considine T., Patel H.A., Anema S.G., Singh H., Creamer L.K. (2007). Interactions of Milk Proteins during Heat and High Hydrostatic Pressure Treatments—A Review. Innov. Food Sci. Emerg. Technol..

[14] Visschers R.W., de Jongh H.H.J. (2005). Disulphide Bond Formation in Food Protein Aggregation and Gelation. Biotechnol. Adv..

[15] Boukil A., Suwal S., Chamberland J., Pouliot Y., Doyen A. (2018). Ultrafiltration Performance and Recovery of Bioactive Peptides after Fractionation of Tryptic Hydrolysate Generated from Pressure-Treated β-Lactoglobulin. J. Membr. Sci..

[16] Varunjikar M.S., Belghit I., Gjerde J., Palmblad M., Oveland E., Rasinger J.D. (2022). Shotgun proteomics approaches for authentication, biological analyses, and allergen detection in feed and food-grade insect species. Food Control.

[17] Giulia L., Tullia T., Pratesi F., Claudia F., Puxeddu I., Migliorini P., Natasja G., Johan J., Stefaan D., Caligiani A. (2020). Shotgun Proteomics, in-Silico Evaluation and Immunoblotting Assays for Allergenicity Assessment of Lesser Mealworm, Black Soldier Fly and Their Protein Hydrolysates. Sci. Rep..

[18] Yi L., Van Boekel M.A., Boeren S., Lakemond C.M. (2016). Protein Identification and in Vitro Digestion of Fractions from *Tenebrio Molitor*. Eur. Food Res. Technol..

[19] de Gier S., Verhoeckx K. (2018). Insect (Food) Allergy and Allergens. Mol. Immunol..

[20] Gravel A., Doyen A. (2020). The Use of Edible Insect Proteins in Food: Challenges and Issues Related to Their Functional Properties. Innov. Food Sci. Emerg. Technol..

[21] Gkinali A.-A., Matsakidou A., Vasileiou E., Paraskevopoulou A. (2022). Potentiality of *Tenebrio Molitor* Larva-Based Ingredients for the Food Industry: A Review. Trends Food Sci. Technol..

[22] Kim T.-K., Yong H.I., Kang M.-C., Jung S., Jang H.W., Choi Y.-S. (2021). Effects of High Hydrostatic Pressure on Technical Functional Properties of Edible Insect Protein. Food Sci. Anim. Resour..

[23] Ugur A.E., Bolat B., Oztop M.H., Alpas H. (2021). Effects of High Hydrostatic Pressure (HHP) Processing and Temperature on Physicochemical Characterization of Insect Oils Extracted from Acheta Domesticus (*House cricket*) and *Tenebrio Molitor* (Yellow Mealworm). Waste Biomass Valor.

[24] Bolat B., Ugur A.E., Oztop M.H., Alpas H. (2021). Effects of High Hydrostatic Pressure Assisted Degreasing on the Technological Properties of Insect Powders Obtained from Acheta Domesticus & *Tenebrio Molitor*. J. Food Eng..

[25] Yi L., Lakemond C.M., Sagis L.M., Eisner-Schadler V., van Huis A., van Boekel M.A. (2013). Extraction and Characterisation of Protein Fractions from Five Insect Species. Food Chem..

[26] Wessels M.L.J., Azzollini D., Fogliano V. (2020). Frozen Storage of Lesser Mealworm Larvae (*Alphitobius diaperinus*) Changes Chemical Properties and Functionalities of the Derived Ingredients. Food Chem..

[27] Möller M., Denicola A. (2002). Protein tryptophan accessibility studied by fluorescence quenching. Biochem. Mol. Biol. Educ..

[28] Mark B.L., McKenna S.A., Khajehpour M., Moo-Young M. (2011). 1.11—Protein Structural Analysis. Comprehensive Biotechnology.

[29] Nakai S. (2003). Measurement of Protein Hydrophobicity. Curr. Protoc. Food Anal. Chem..

[30] Azagoh C., Ducept F., Garcia R., Rakotozafy L., Cuvelier M.-E., Keller S., Lewandowski R., Mezdour S. (2016). Extraction and Physicochemical Characterization of *Tenebrio Molitor* Proteins. Food Res. Int..

[31] Marciniak A., Suwal S., Brisson G., Britten M., Pouliot Y., Doyen A. (2018). Studying a Chaperone-like Effect of Beta-Casein on Pressure-Induced Aggregation of Beta-Lactoglobulin in the Presence of Alpha-Lactalbumin. Food Hydrocoll..

[32] Chao D., He R., Jung S., Aluko R.E. (2013). Effect of Pressure or Temperature Pretreatment of Isolated Pea Protein on Properties of the Enzymatic Hydrolysates. Food Res. Int..

[33] Hayakawa S., Nakai S. (2006). Relationships of Hydrophobicity and Net Charge to the Solubility of Milk and Soy Proteins. J. Food Sci..

[34] Chen J., Mu T., Zhang M., Goffin D., Sun H., Ma M., Liu X., Zhang D. (2018). Structure, Physicochemical, and Functional Properties of Protein Isolates and Major Fractions from Cumin (*Cuminum cyminum*) Seeds. Int. J. Food Prop..

[35] Lundgren D.H., Hwang S.-I., Wu L., Han D.K. (2010). Role of Spectral Counting in Quantitative Proteomics. Expert Rev. Proteom..

[36] Yong Y.H., Foegeding E.A. (2010). Caseins: Utilizing Molecular Chaperone Properties to Control Protein Aggregation in Foods. J. Agric. Food Chem..

[37] de Souza H.K.S., Bai G., Gonçalves M.D.P., Bastos M. (2009). Whey Protein Isolate–Chitosan Interactions: A Calorimetric and Spectroscopy Study. Thermochim. Acta.

[38] Hsu K.-C., Ko W.-C. (2001). Effect of Hydrostatic Pressure on Aggregation and Viscoelastic Properties of Tilapia (*Orechromis niloticus*) Myosin. J. Food Sci..

[39] Kanno C., Mu T.-H., Hagiwara T., Ametani M., Azuma N. (1998). Gel Formation from Industrial Milk Whey Proteins under Hydrostatic Pressure:  Effect of Hydrostatic Pressure and Protein Concentration. J. Agric. Food Chem..

[40] Van der Plancken I., Van Loey A., Hendrickx M.E.G. (2005). Changes in Sulfhydryl Content of Egg White Proteins Due to Heat and Pressure Treatment. J. Agric. Food Chem..

[41] Wang J.-M., Yang X.-Q., Yin S.-W., Zhang Y., Tang C.-H., Li B.-S., Yuan D.-B., Guo J. (2011). Structural Rearrangement of Ethanol-Denatured Soy Proteins by High Hydrostatic Pressure Treatment. J Agric. Food Chem..

[42] Zhang Z., Li Y., Lee M.C., Ravanfar R., Padilla-Zakour O.I., Abbaspourrad A. (2020). The Impact of High-Pressure Processing on the Structure and Sensory Properties of Egg White-Whey Protein Mixture at Acidic Conditions. Food Bioprocess Technol..

[43] David-Birman T., Raften G., Lesmes U. (2018). Effects of Thermal Treatments on the Colloidal Properties, Antioxidant Capacity and in-Vitro Proteolytic Degradation of Cricket Flour. Food Hydrocoll..

[44] Zhang Y., Zhang X., Zhang Z., Chen Z., Jing X., Wang X. (2022). Effect of High Hydrostatic Pressure Treatment on the Structure and Physicochemical Properties of Millet Gliadin. LWT.

[45] Perreault V., Hénaux L., Bazinet L., Doyen A. (2017). Pretreatment of Flaxseed Protein Isolate by High Hydrostatic Pressure: Impacts on Protein Structure, Enzymatic Hydrolysis and Final Hydrolysate Antioxidant Capacities. Food Chem..

[46] Patel H.A., Singh H., Anema S.G., Creamer L.K. (2006). Effects of Heat and High Hydrostatic Pressure Treatments on Disulfide Bonding Interchanges among the Proteins in Skim Milk. J. Agric. Food Chem..

[47] Patel H.A., Singh H., Havea P., Considine T., Creamer L.K. (2005). Pressure-Induced Unfolding and Aggregation of the Proteins in Whey Protein Concentrate Solutions. J. Agric. Food Chem..

[48] Luo L., Zhang R., Palmer J., Hemar Y., Yang Z. (2021). Impact of High Hydrostatic Pressure on the Gelation Behavior and Microstructure of Quinoa Protein Isolate Dispersions. ACS Food Sci. Technol..

[49] Bhattacharjee C., Das K.P. (2000). Thermal Unfolding and Refolding of β-Lactoglobulin. Eur. J. Biochem..

[50] Li S., Li L., Zhu X., Ning C., Cai K., Zhou C. (2019). Conformational and Charge Changes Induced by L-Arginine and l-Lysine Increase the Solubility of Chicken Myosin. Food Hydrocoll..

[51] Acero-Lopez A., Ullah A., Offengenden M., Jung S., Wu J. (2012). Effect of High Pressure Treatment on Ovotransferrin. Food Chem..

[52] Qu W., Zhang X., Han X., Wang Z., He R., Ma H. (2018). Structure and Functional Characteristics of Rapeseed Protein Isolate-Dextran Conjugates. Food Hydrocoll..

[53] Queirós R.P., Saraiva J.A., da Silva J.A.L. (2018). Tailoring Structure and Technological Properties of Plant Proteins Using High Hydrostatic Pressure. Crit. Rev. Food Sci. Nutr..

[54] Puppo C., Chapleau N., Speroni F., de Lamballerie-Anton M., Michel F., Añón C., Anton M. (2004). Physicochemical Modifications of High-Pressure-Treated Soybean Protein Isolates. J. Agric. Food Chem..

[55] Qin Z., Guo X., Lin Y., Chen J., Liao X., Hu X., Wu J. (2013). Effects of High Hydrostatic Pressure on Physicochemical and Functional Properties of Walnut (*Juglans regia* L.) Protein Isolate. J. Sci. Food Agric..

[56] Lee H., Kim J., Ji D., Lee C. (2019). Effects of Heating Time and Temperature on Functional Properties of Proteins of Yellow Mealworm Larvae (*Tenebrio molitor* L.). Food Sci. Anim. Resour..

[57] Rivero-Pino F., Espejo-Carpio F.J., Pérez-Gálvez R., Guadix A., Guadix E.M. (2020). Effect of Ultrasound Pretreatment and Sequential Hydrolysis on the Production of *Tenebrio Molitor* Antidiabetic Peptides. Food Bioprod. Processing.

[58] Huppertz T., Fox P.F., Kelly A.L. (2004). High Pressure Treatment of Bovine Milk: Effects on Casein Micelles and Whey Proteins. J. Dairy Res..

[59] Jegouic M., Grinberg V.Y., Guingant A., Haertlé T. (1997). Baric Oligomerization in α-Lactalbumin/β-Lactoglobulin Mixtures. J. Agric. Food Chem..

[60] Hagner-Holler S., Schoen A., Erker W., Marden J.H., Rupprecht R., Decker H., Burmester T. (2004). A Respiratory Hemocyanin from an Insect. Proc. Natl. Acad. Sci. USA.

[61] Burmester T., Schellen K. (1996). Common Origin of Arthropod Tyrosinase, Arthropod Hemocyanin, Insect Hexamerin, and Dipteran Arylphorin Receptor. J. Mol. Evol..

[62] Zhang Y., Deng Y., Zhao Y. (2017). Structure-Based Modelling of Hemocyanin Allergenicity in Squid and Its Response to High Hydrostatic Pressure. Sci. Rep..

[63] Reinhart G., Gratton E., Mantulin W.W., Winter R., Jonas J. (1993). Dissociation of Large Oligomeric Proteins by High Hydrostatic Pressure: Dynamic Light Scattering Studies. High Pressure Chemistry, Biochemistry and Materials Science.

[64] Grauwet T., der Plancken I.V., Vervoort L., Hendrickx M.E., Loey A.V. (2009). Investigating the Potential of Bacillus Subtilis α-Amylase as a Pressure-Temperature-Time Indicator for High Hydrostatic Pressure Pasteurization Processes. Biotechnol. Prog..

[65] Gomes M.R.A., Clark R., Ledward D.A. (1998). Effects of High Pressure on Amylases and Starch in Wheat and Barley Flours. Food Chem..

[66] Krüger M., Linke W.A. (2011). The Giant Protein Titin: A Regulatory Node That Integrates Myocyte Signaling Pathways. J. Biol. Chem..

[67] Rivalain N., Roquain J., Demazeau G. (2010). Development of High Hydrostatic Pressure in Biosciences: Pressure Effect on Biological Structures and Potential Applications in Biotechnologies. Biotechnol. Adv..

[68] Murchie L.W., Cruz-Romero M., Kerry J.P., Linton M., Patterson M.F., Smiddy M., Kelly A.L. (2005). High Pressure Processing of Shellfish: A Review of Microbiological and Other Quality Aspects. Innov. Food Sci. Emerg. Technol..

[69] Ko W.-C., Hwang J.-S., Jao C.-L., Hsu K.-C. (2004). Denaturation of Tilapia Myosin Fragments by High Hydrostatic Pressure. J. Food Sci..

[70] Ko W.C., Jao C.L., Hsu K.C. (2003). Effect of Hydrostatic Pressure on Molecular Conformation of Tilapia (*Orechromis niloticus*) Myosin. J. Food Sci..

[71] Hsu K.-C., Hwang J.-S., Yu C.-C., Jao C.-L. (2007). Changes in Conformation and in Sulfhydryl Groups of Actomyosin of Tilapia (Orechromis Niloticus) on Hydrostatic Pressure Treatment. Food Chem..

